# Supplementary material for: Whole Sequencing and Detailed Analysis of SARS-CoV-2 Genomes in Southeast Spain: Identification of Recurrent Mutations in the 20E (EU1) Variant with Some Clinical Implications
Source: Diseases. 2023 Mar 31;11(2):54. doi: 10.3390/diseases11020054 (PMC10123601; doi:10.3390/diseases11020054)
Supplement: Supplementary file 1 [file diseases-11-00054-s001.zip › Figure S1.pdf]

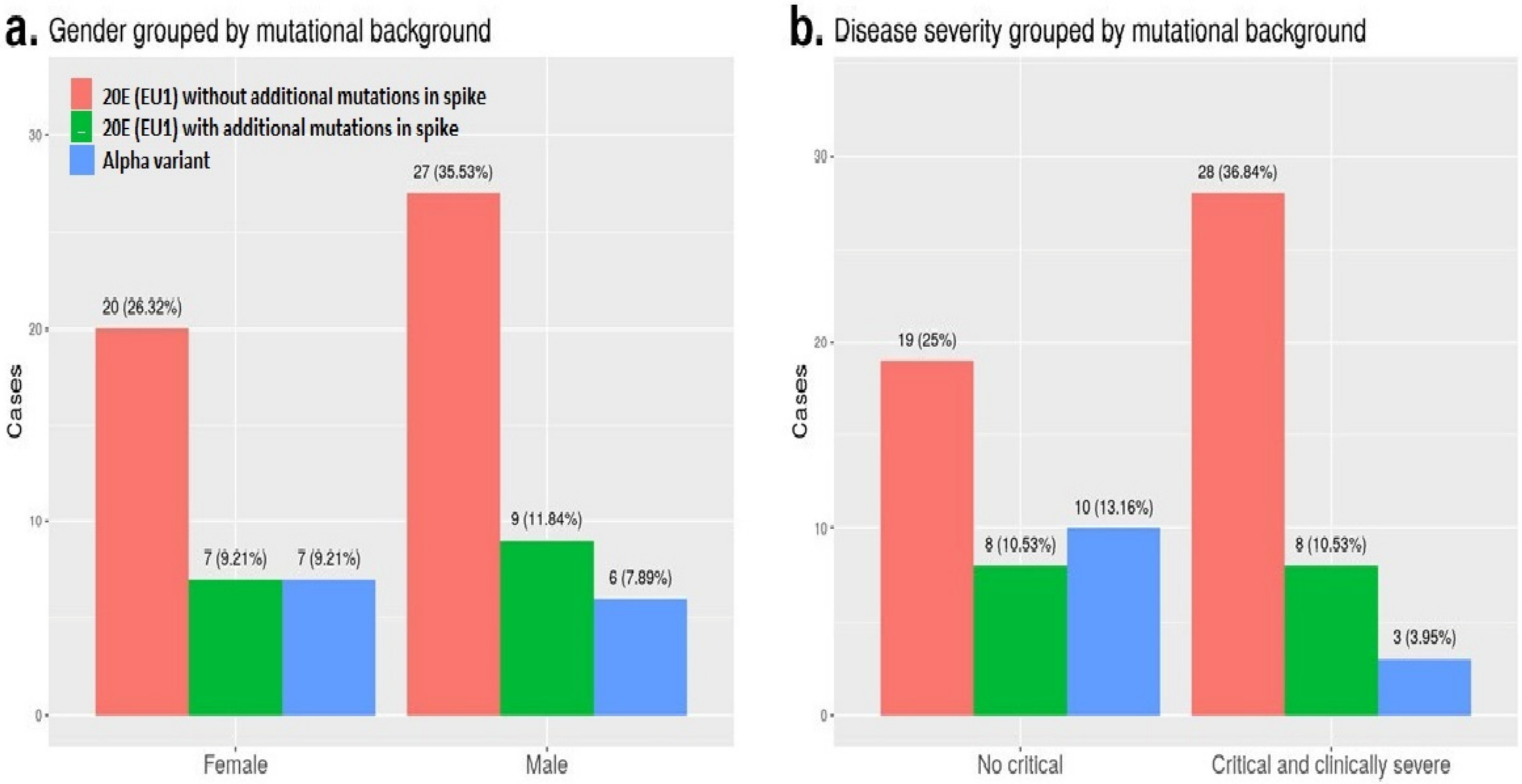

Figure S1. Gender (**a**) and disease severity (**b**) of 76 patients grouped by mutational background. According to the Pearson's chi-square, relationship between the nominal variables with $\chi^2(2) = 0.53$, $r > 0.05$ (gender versus mutational background and $\chi^2(2) = 5.44$, $r > 0.05$ (disease severity versus mutational background) could not be found.
