# Supplementary material for: Whole Sequencing and Detailed Analysis of SARS-CoV-2 Genomes in Southeast Spain: Identification of Recurrent Mutations in the 20E (EU1) Variant with Some Clinical Implications
Source: Diseases. 2023 Mar 31;11(2):54. doi: 10.3390/diseases11020054 (PMC10123601; doi:10.3390/diseases11020054)
Supplement: Supplementary file 1 [file diseases-11-00054-s001.zip › Figure S2.pdf]

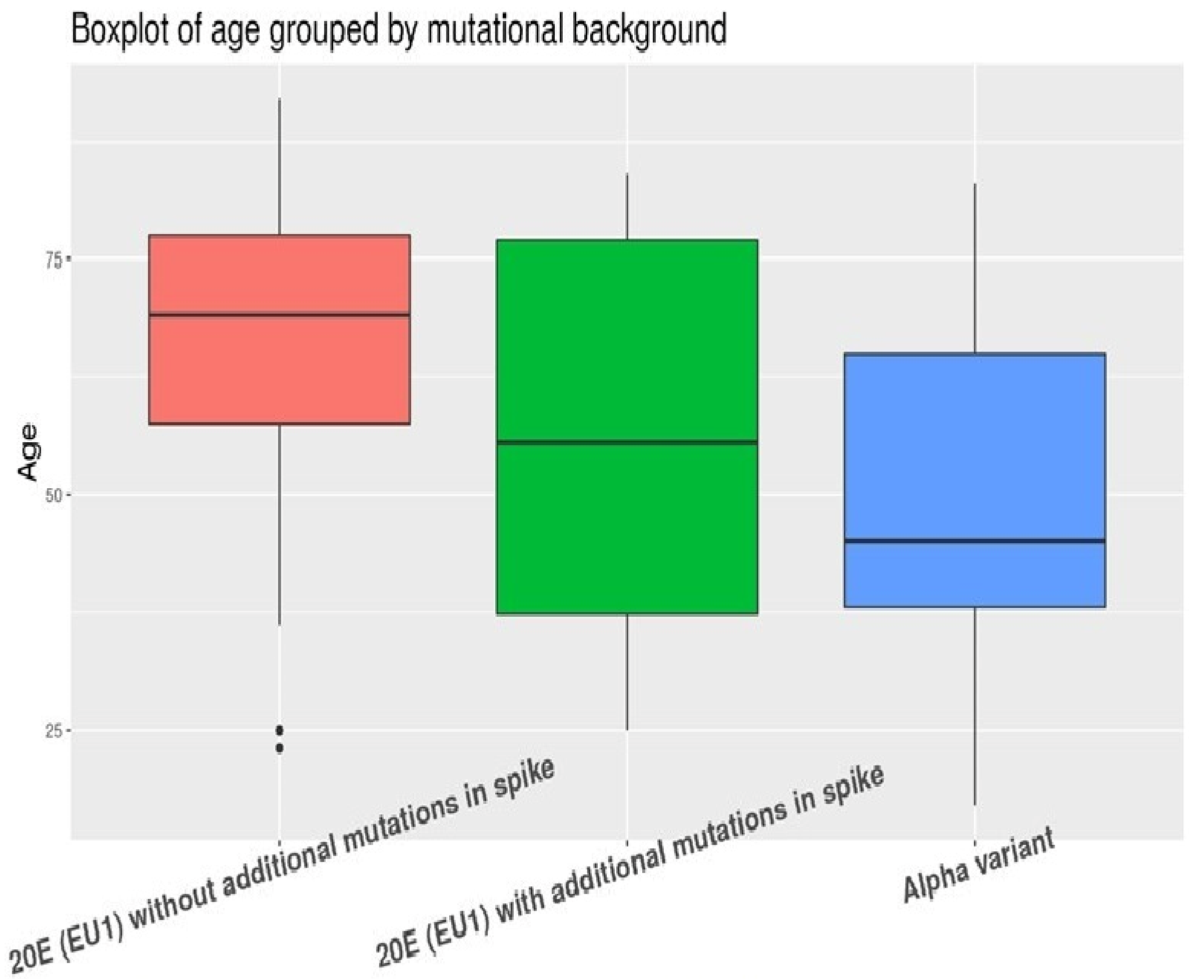


Figure S2. Bloxplot of age grouped by mutational background. The one-way ANOVA followed by multiple comparisons with Bonferroni correction revealed significant differences between the 20E (EU1) cases without additional mutations in spike and the Alpha variant group. In each box, the median is marked as a horizontal line, and Q1 and Q3 asvertical ones.
