# Supplementary material for: Whole Sequencing and Detailed Analysis of SARS-CoV-2 Genomes in Southeast Spain: Identification of Recurrent Mutations in the 20E (EU1) Variant with Some Clinical Implications
Source: Diseases. 2023 Mar 31;11(2):54. doi: 10.3390/diseases11020054 (PMC10123601; doi:10.3390/diseases11020054)
Supplement: Supplementary file 1 [file diseases-11-00054-s001.zip › Table S1.pdf]

Table S1 (Supplementary). Detailed information of the 88 SARS-CoV-2 sequenced genomes belonging to each sample taken from COVID-19 patients between October 2020 and April 2021 at the "Reina Sofía" Hospital (Murcia, Spain). Along the GISAID Accession ID and the SARS-CoV-2 variants detected in the study, the sequencing coverage and the percentage of sequenced bases are indicated in addition to the clinical data of the patients.

Sample name	GISAID Accession ID	Nexstrain Clade	% of non-N bases	Median Coverage	Collection date of the sample	Gender	Patient age	Disease severity (WHO 1-7 scale)
RS15	EPI_ISL_11540720	20E (EU1)	99.71%	1,360x	2021-01-15	Female	69	5
RS16	EPI_ISL_11586307	20E (EU1)	99.72%	1,265x	2021-01-18	Male	72	7
RS17	EPI_ISL_11620933	20E (EU1)	99.74%	1,873x	2021-01-29	Male	58	6
RS18	EPI_ISL_11621374	20E (EU1)	98.98%	1,383x	2020-10-25	Male	79	7
RS19	EPI_ISL_11621478	20E (EU1)	99.71%	1,715x	2021-01-18	Female	84	7
RS20	EPI_ISL_11621901	20E (EU1)	99.72%	1,359x	2020-12-28	Male	86	7
RS21	EPI_ISL_11623510	20E (EU1)	98.19%	1,313x	2021-01-07	Female	77	5
RS22	EPI_ISL_11623527	20E (EU1)	99.72%	1,588x	2021-01-28	Male	46	5
RS23	EPI_ISL_11623867	20E (EU1)	99.72%	1,741x	2021-02-11	Female	77	7
RS24	EPI_ISL_11623868	20I (Alpha, V1)	99.70%	1,099x	2021-02-24	Male	82	7
RS25	EPI_ISL_11634150	20E (EU1)	99.71%	1,404x	2020-12-12	Male	53	4
RS26	EPI_ISL_11634151	20E (EU1)	99.72%	1,486x	2020-12-18	Female	58	4
RS27	EPI_ISL_11634319	20E (EU1)	99.74%	1,546x	2020-12-31	Male	77	1
RS28	EPI_ISL_11635063	20A	99.72%	1,825x	2021-01-01	Female	65	3
RS29	EPI_ISL_11635064	20E (EU1)	99.72%	1,544x	2021-01-11	Female	47	1
RS30	EPI_ISL_11635065	20E (EU1)	99.71%	1,628x	2021-01-17	Female	75	4
RS31	EPI_ISL_11635067	20E (EU1)	99.72%	1,824x	2021-02-09	Male	91	3
RS32	EPI_ISL_11635200	20I (Alpha, V1)	99.72%	1,815x	2021-02-10	Male	83	4
RS33	EPI_ISL_11635217	20E (EU1)	99.72%	1,757x	2021-01-04	Female	78	4
RS34	EPI_ISL_11635219	20E (EU1)	84.45%	303x	2021-03-04	Male	84	4
RS35	EPI_ISL_11635237	20E (EU1)	98.89%	900x	2021-03-11	Female	76	5
RS36	EPI_ISL_11637549	20I (Alpha, V1)	99.72%	1,643x	2021-03-13	Female	17	1
RS37	EPI_ISL_11643426	20I (Alpha, V1)	99.72%	1,533x	2021-03-23	Female	63	2
RS38	EPI_ISL_11646217	20E (EU1)	99.72%	1,338x	2021-03-29	Female	80	4
RS39	EPI_ISL_11684895	20I (Alpha, V1)	99.75%	1,565x	2021-03-30	Male	38	1
RS40	EPI_ISL_11685202	20E (EU1)	99.73%	1,355x	2021-04-05	Male	40	2
RS41	EPI_ISL_11685203	20A/S:98F	99.73%	875x	2021-01-25	Female	63	7

RS42	EPI_ISL_11685204	20E (EU1)	99.71%	1,559x	2021-01-15	Male	77	7
RS43	EPI_ISL_11685205	20E (EU1)	98.98%	1,211x	2021-01-27	Male	61	7
RS44	EPI_ISL_11688802	20E (EU1)	99.72%	1,463x	2021-01-27	Female	57	5
RS45	EPI_ISL_11690593	20E (EU1)	82.13%	506x	2021-01-29	Male	69	7
RS46	EPI_ISL_11693524	20E (EU1)	99.71%	1,206x	2021-01-29	Male	86	7
RS47	EPI_ISL_11696353	20E (EU1)	99.74%	1,312x	2021-02-04	Male	71	7
RS48	EPI_ISL_11696722	20E (EU1)	99.72%	1,064x	2021-01-24	Female	77	4
RS49	EPI_ISL_11696732	20E (EU1)	99.70%	1,390x	2021-01-29	Male	84	5
RS50	EPI_ISL_11696733	20E (EU1)	99.71%	1,147x	2021-02-03	Male	75	4
RS51	EPI_ISL_11696748	20I (Alpha, V1)	99.72%	1,380x	2021-02-19	Female	65	4
RS52	EPI_ISL_11696749	20E (EU1)	99.74%	1,352x	2021-03-23	Female	80	4
RS53	EPI_ISL_11696750	20I (Alpha, V1)	99.73%	1,418x	2021-03-24	Female	36	1
RS54	EPI_ISL_11696751	20E (EU1)	99.72%	1,377x	2021-01-04	Male	23	1
RS55	EPI_ISL_11696752	20E (EU1)	99.76%	1,448x	2021-01-05	Female	36	1
RS56	EPI_ISL_11728097	20E (EU1)	99.72%	1,341x	2021-01-05	Female	27	1
RS57	EPI_ISL_11729677	20A/S:98F	99.75%	1,382x	2021-01-05	Female	31	1
RS58	EPI_ISL_11731165	20E (EU1)	99.72%	1,585x	2021-01-12	Male	60	1
RS59	EPI_ISL_11754781	20E (EU1)	99.72%	1,345x	2021-01-12	Female	68	2
RS60	EPI_ISL_11754782	20E (EU1)	99.71%	1,302x	2021-01-13	Female	50	1
RS61	EPI_ISL_11754909	20E (EU1)	99.72%	1,517x	2021-01-14	Female	25	4
RS62	EPI_ISL_11755017	20E (EU1)	99.72%	1,295x	2021-01-14	Female	61	2
RS63	EPI_ISL_11755804	20E (EU1)	99.72%	1,273x	2021-01-15	Male	45	3
RS64	EPI_ISL_11757369	20E (EU1)	99.73%	1,337x	2021-01-16	Male	86	4
RS65	EPI_ISL_11758399	20A/S:98F	99.72%	1,416x	2021-01-19	Male	81	3
RS66	EPI_ISL_11760445	20A	99.72%	1,063x	2021-01-19	Female	47	4
RS67	EPI_ISL_11760681	20E (EU1)	99.71%	1,239x	2021-01-19	Male	35	3
RS68	EPI_ISL_11765428	20E (EU1)	99.71%	1,333x	2021-01-19	Male	25	1
RS69	EPI_ISL_11765429	20E (EU1)	99.71%	1,343x	2021-01-19	Male	25	1
RS70	EPI_ISL_11765430	20E (EU1)	99.71%	1,198x	2021-02-01	Female	72	4
RS71	EPI_ISL_11767043	20E (EU1)	99.35%	1,382x	2021-01-25	Male	49	1
RS72	EPI_ISL_11767044	20I (Alpha, V1)	99.73%	1,253x	2021-04-10	Male	45	1
RS73	EPI_ISL_11767045	20E (EU1)	99.72%	1,320x	2021-04-12	Male	38	3

RS74	EPI_ISL_11767046	20J (Gamma, V3)	99.75%	1,511x	2021-04-12	Female	52	1
RS75	EPI_ISL_11767138	20I (Alpha, V1)	99,72%	1,333x	2021-04-12	Female	28	1
RS76	EPI_ISL_11767139	20I (Alpha, V1)	99.72%	1,514x	2021-04-12	Female	43	1
RS77	EPI_ISL_11767140	20I (Alpha, V1)	99.73%	1,547x	2021-04-12	Male	44	1
RS78	EPI_ISL_11767141	21H	99.72%	1,411x	2021-04-12	Male	23	1
RS79	EPI_ISL_11767142	20I (Alpha, V1)	99.72%	1,480x	2021-04-12	Female	45	1
RS81	EPI_ISL_11767143	20E (EU1)	99.72%	1,376x	2021-02-07	Male	59	1
RS82	EPI_ISL_11767144	20E (EU1)	99.72%	1,524x	2021-01-26	Male	58	1
RS83	EPI_ISL_11767145	20E (EU1)	99.71%	1,272x	2021-01-19	Female	53	1
RS84	EPI_ISL_11767147	20A	99.72%	1,325x	2021-01-20	Female	40	1
RS85	EPI_ISL_11768305	20E (EU1)	99.71%	1,250x	2020-10-30	Male	41	6
RS86	EPI_ISL_11778143	20E (EU1)	99.73%	1,447x	2020-12-31	Male	74	1
RS87A	EPI_ISL_11780128	20E (EU1)	99.72%	1,342x	2021-01-01	Male	67	7
RS88	EPI_ISL_11780263	20A/S:98F	99.73%	1,212x	2021-01-05	Male	75	1
RS89	EPI_ISL_11780447	20E (EU1)	99.72%	1,379x	2021-01-20	Male	71	1
RS90	EPI_ISL_11780673	20I (Alpha, V1)	99.75%	1,222x	2021/02/07	Male	68	1
RS91	EPI_ISL_11781250	20E (EU1)	99.72%	1,180x	2020/10/20	Female	85	4
RS92	EPI_ISL_11781612	20E (EU1)	97.68%	1,263x	2020/10/17	Male	59	5
RS93	EPI_ISL_11782759	20E (EU1)	98.95%	1,761x	2021/01/27	Female	51	1
RS94	EPI_ISL_11784816	20E (EU1)	98.85%	1,321x	2021/02/04	Male	76	6
RS95	EPI_ISL_11785652	20A/S:98F	99.35%	1,328x	2021/01/21	Male	52	5
RS96	EPI_ISL_11793412	20E (EU1)	98.39%	1,152x	2021/02/10	Female	92	4
RS97	EPI_ISL_11793789	20E (EU1)	99.32%	1,477x	2021/01/28	Male	80	1
RS98	EPI_ISL_11794338	20A/S:98F	97.52%	1,190x	2020/10/14	Female	89	3
RS99	EPI_ISL_11794355	20E (EU1)	99.47%	1,245x	2021/02/02	Female	69	5
RS100	EPI_ISL_11794357	20C	98.57%	1,369x	2020/10/29	Male	55	4
RS101	EPI_ISL_11794379	20E (EU1)	98.70%	1,726x	2020/10/17	Male	29	1
RS102	EPI_ISL_11794519	20E (EU1)	99.71%	1,605x	2021/01/19	Female	83	1
RS103	EPI_ISL_11794610	20E (EU1)	98.20%	867x	2020/10/21	Female	60	2
