# Supplementary material for: Whole Sequencing and Detailed Analysis of SARS-CoV-2 Genomes in Southeast Spain: Identification of Recurrent Mutations in the 20E (EU1) Variant with Some Clinical Implications
Source: Diseases. 2023 Mar 31;11(2):54. doi: 10.3390/diseases11020054 (PMC10123601; doi:10.3390/diseases11020054)
Supplement: Supplementary file 1 [file diseases-11-00054-s001.zip › Table S2.pdf]

Table S2 (Supplementary). Detailed information of the 88 SARS-CoV-2 sequenced genomes belonging to each sample taken from COVID-19 patients between October 2020 and April 2021 at the "Reina Sofía" Hospital (Murcia, Spain). Along the GISAID Accession ID and the SARS-CoV-2 variants detected in the study, the number of nucleotide variants is indicated on the total genome and the spike gene with regard to the reference genome (SNVs: Single Nucleotide Variants).

Sample name	GISAID Accession ID	Nexstrain Clade	Total number of significant nucleotide variants along with its percentage on the whole genome				Total number of significant nucleotide variants and its percentage within spike gene			
			SNVs	Deletions	Insertions	%	SNVs	Deletions	Insertions	%
RS15	EPI_ISL_11540720	20E (EU1)	17	0	0	0.057	2	0	0	0.052
RS16	EPI_ISL_11586307	20E (EU1)	20	0	0	0.067	2	0	0	0.052
RS17	EPI_ISL_11620933	20E (EU1)	20	0	0	0.067	2	0	0	0.052
RS18	EPI_ISL_11621374	20E (EU1)	15	0	0	0.050	2	0	0	0.052
RS19	EPI_ISL_11621478	20E (EU1)	18	0	0	0.060	2	0	0	0.052
RS20	EPI_ISL_11621901	20E (EU1)	18	0	0	0.060	3	0	0	0.079
RS21	EPI_ISL_11623510	20E (EU1)	19	0	0	0.064	2	0	0	0.052
RS22	EPI_ISL_11623527	20E (EU1)	20	0	0	0.067	3	0	0	0.079
RS23	EPI_ISL_11623867	20E (EU1)	20	0	0	0.067	3	0	0	0.079
RS24	EPI_ISL_11623868	20I (Alpha, V1)	30	19	1	0.167	7	9	0	0.419
RS25	EPI_ISL_11634150	20E (EU1)	22	0	0	0.074	4	0	0	0.105
RS26	EPI_ISL_11634151	20E (EU1)	17	0	0	0.057	3	0	0	0.079
RS27	EPI_ISL_11634319	20E (EU1)	16	0	0	0.054	2	0	0	0.052
RS28	EPI_ISL_11635063	20A	17	0	0	0.057	1	0	0	0.026
RS29	EPI_ISL_11635064	20E (EU1)	21	2	0	0.077	2	0	0	0.052
RS30	EPI_ISL_11635065	20E (EU1)	17	3	0	0.067	2	0	0	0.052
RS31	EPI_ISL_11635067	20E (EU1)	20	0	0	0.067	3	0	0	0.079
RS32	EPI_ISL_11635200	20I (Alpha, V1)	30	19	0	0.164	8	9	0	0.445
RS33	EPI_ISL_11635217	20E (EU1)	19	0	0	0.064	2	0	0	0.052
RS34	EPI_ISL_11635219	20E (EU1)	18	2	0	0.067	4	0	0	0.105
RS35	EPI_ISL_11635237	20E (EU1)	24	0	0	0.080	5	0	0	0.131
RS36	EPI_ISL_11637549	20I (Alpha, V1)	36	19	0	0.184	7	9	0	0.419
RS37	EPI_ISL_11643426	20I (Alpha, V1)	33	19	0	0.174	7	9	0	0.419
RS38	EPI_ISL_11646217	20E (EU1)	19	0	0	0.064	5	0	0	0.131
RS39	EPI_ISL_11684895	20I (Alpha, V1)	34	19	0	0.177	8	9	0	0.445

RS40	EPI_ISL_11685202	20E (EU1)	31	16	0	0.157	5	6	0	0.288
RS41	EPI_ISL_11685203	20A/S:98F	15	0	0	0.050	2	0	0	0.052
RS42	EPI_ISL_11685204	20E (EU1)	20	0	0	0.067	3	0	0	0.079
RS43	EPI_ISL_11685205	20E (EU1)	17	0	0	0.057	2	0	0	0.052
RS44	EPI_ISL_11688802	20E (EU1)	20	0	0	0.067	2	0	0	0.052
RS45	EPI_ISL_11690593	20E (EU1)	17	0	0	0.057	2	0	0	0.052
RS46	EPI_ISL_11693524	20E (EU1)	17	0	0	0.057	2	0	0	0.052
RS47	EPI_ISL_11696353	20E (EU1)	18	0	0	0.060	2	0	0	0.052
RS48	EPI_ISL_11696722	20E (EU1)	20	0	0	0.067	4	0	0	0.105
RS49	EPI_ISL_11696732	20E (EU1)	18	0	0	0.060	2	0	0	0.052
RS50	EPI_ISL_11696733	20E (EU1)	17	4	0	0.070	3	0	0	0.079
RS51	EPI_ISL_11696748	20I (Alpha, V1)	30	19	0	0.164	8	9	0	0.445
RS52	EPI_ISL_11696749	20E (EU1)	19	0	0	0.064	5	0	0	0.131
RS53	EPI_ISL_11696750	20I (Alpha, V1)	35	19	0	0.181	8	9	0	0.445
RS54	EPI_ISL_11696751	20E (EU1)	18	0	0	0.060	2	0	0	0.052
RS55	EPI_ISL_11696752	20E (EU1)	21	0	0	0.070	2	0	0	0.052
RS56	EPI_ISL_11728097	20E (EU1)	19	0	0	0.064	4	0	0	0.105
RS57	EPI_ISL_11729677	20A/S:98F	17	0	0	0.057	2	0	0	0.052
RS58	EPI_ISL_11731165	20E (EU1)	19	0	0	0.064	2	0	0	0.052
RS59	EPI_ISL_11754781	20E (EU1)	20	0	0	0.067	3	0	0	0.079
RS60	EPI_ISL_11754782	20E (EU1)	21	0	0	0.070	3	0	0	0.079
RS61	EPI_ISL_11754909	20E (EU1)	23	0	0	0.077	4	0	0	0.105
RS62	EPI_ISL_11755017	20E (EU1)	19	0	0	0.064	4	0	0	0.105
RS63	EPI_ISL_11755804	20E (EU1)	19	0	0	0.064	4	0	0	0.105
RS64	EPI_ISL_11757369	20E (EU1)	18	0	0	0.060	2	0	0	0.052
RS65	EPI_ISL_11758399	20A/S:98F	15	0	0	0.050	2	0	0	0.052
RS66	EPI_ISL_11760445	20A	26	0	0	0.087	5	0	0	0.131
RS67	EPI_ISL_11760681	20E (EU1)	18	9	0	0.090	3	0	0	0.079
RS68	EPI_ISL_11765428	20E (EU1)	20	0	0	0.067	3	0	0	0.079
RS69	EPI_ISL_11765429	20E (EU1)	20	0	0	0.067	3	0	0	0.079
RS70	EPI_ISL_11765430	20E (EU1)	24	0	0	0.080	2	0	0	0.052
RS71	EPI_ISL_11767043	20E (EU1)	21	0	0	0.070	2	0	0	0.052

RS72	EPI_ISL_11767044	20I (Alpha, V1)	38	19	0	0.191	7	9	0	0.419
RS73	EPI_ISL_11767045	20E (EU1)	33	10	0	0.144	5	0	0	0.131
RS74	EPI_ISL_11767046	20J (Gamma, V3)	38	9	4	0.171	13	0	0	0.340
RS75	EPI_ISL_11767138	20I (Alpha, V1)	29	19	0	0.161	7	9	0	0.419
RS76	EPI_ISL_11767139	20I (Alpha, V1)	36	19	0	0.184	7	9	0	0.419
RS77	EPI_ISL_11767140	20I (Alpha, V1)	40	19	0	0.197	7	9	0	0.419
RS78	EPI_ISL_11767141	21H	33	10	3	0.154	9	0	3	0.314
RS79	EPI_ISL_11767142	20I (Alpha, V1)	40	19	0	0.197	7	9	0	0.419
RS81	EPI_ISL_11767143	20E (EU1)	22	0	0	0.074	2	0	0	0.052
RS82	EPI_ISL_11767144	20E (EU1)	21	0	0	0.070	4	0	0	0.105
RS83	EPI_ISL_11767145	20E (EU1)	20	0	0	0.067	3	0	0	0.079
RS84	EPI_ISL_11767147	20A	18	0	0	0.060	2	0	0	0.052
RS85	EPI_ISL_11768305	20E (EU1)	16	0	0	0.054	2	0	0	0.052
RS86	EPI_ISL_11778143	20E (EU1)	20	0	0	0.067	2	0	0	0.052
RS87A	EPI_ISL_11780128	20E (EU1)	18	0	0	0.060	2	0	0	0.052
RS88	EPI_ISL_11780263	20A/S:98F	19	0	0	0.064	4	0	0	0.105
RS89	EPI_ISL_11780447	20E (EU1)	17	0	0	0.057	2	0	0	0.052
RS90	EPI_ISL_11780673	20I (Alpha, V1)	30	19	0	0.164	8	9	0	0.445
RS91	EPI_ISL_11781250	20E (EU1)	15	0	0	0.050	2	0	0	0.052
RS92	EPI_ISL_11781612	20E (EU1)	13	0	0	0.043	2	0	0	0.052
RS93	EPI_ISL_11782759	20E (EU1)	19	0	0	0.064	2	0	0	0.052
RS94	EPI_ISL_11784816	20E (EU1)	17	0	0	0.057	2	0	0	0.052
RS95	EPI_ISL_11785652	20A/S:98F	16	0	0	0.054	2	0	0	0.052
RS96	EPI_ISL_11793412	20E (EU1)	16	0	0	0.054	2	0	0	0.052
RS97	EPI_ISL_11793789	20E (EU1)	18	0	0	0.060	2	0	0	0.052
RS98	EPI_ISL_11794338	20A/S:98F	16	0	0	0.054	2	0	0	0.052
RS99	EPI_ISL_11794355	20E (EU1)	17	0	0	0.057	2	0	0	0.052
RS100	EPI_ISL_11794357	20C	20	0	0	0.067	2	0	0	0.052
RS101	EPI_ISL_11794379	20E (EU1)	17	0	0	0.057	4	0	0	0.105
RS102	EPI_ISL_11794519	20E (EU1)	17	0	0	0.057	2	0	0	0.052
RS103	EPI_ISL_11794610	20E (EU1)	14	0	0	.047	2	0	0	0.052
